# Digital Pathology Transformation in a Supraregional Germ Cell Tumour Network

**DOI:** 10.3390/diagnostics11122191

**Published:** 2021-11-25

**Authors:** Richard Colling, Andrew Protheroe, Mark Sullivan, Ruth Macpherson, Mark Tuthill, Jacqueline Redgwell, Zoe Traill, Angus Molyneux, Elizabeth Johnson, Niveen Abdullah, Andrea Taibi, Nikki Mercer, Harry R. Haynes, Anthony Sackville, Judith Craft, Joao Reis, Gabrielle Rees, Maria Soares, Ian S. D. Roberts, Darrin Siiankoski, Helen Hemsworth, Derek Roskell, Sharon Roberts-Gant, Kieron White, Jens Rittscher, Jim Davies, Lisa Browning, Clare Verrill

**Affiliations:** 1 Nuffield Department of Surgical Sciences, University of Oxford, Oxford OX3 9DU, UK; clare.verrill@ouh.nhs.uk; 2 Department of Cellular Pathology, Oxford University Hospitals NHS Foundation Trust, Oxford OX3 9DU, UK; Anthony.Sackville@ouh.nhs.uk (A.S.); Judith.Craft@ouh.nhs.uk (J.C.); Joao.reis@ouh.nhs.uk (J.R.); Gabrielle.Rees@ouh.nhs.uk (G.R.); Maria.soares@ouh.nhs.uk (M.S.); ian.roberts@ouh.nhs.uk (I.S.D.R.); darrin.siiankoski@ouh.nhs.uk (D.S.); helen.hemsworth@ouh.nhs.uk (H.H.); derek.roskell@ouh.nhs.uk (D.R.); sharon.roberts-gant@ouh.nhs.uk (S.R.-G.); Kieron.white@ouh.nhs.uk (K.W.); lisa.browning@ouh.nhs.uk (L.B.); 3 Department of Oncology, University of Oxford, Oxford OX3 9DU, UK; Andrew.Protheroe@ouh.nhs.uk; 4 Oxford Cancer Centre, Oxford University Hospitals NHS Foundation Trust, Oxford OX3 9DU, UK; 5 Urology Department, Oxford University Hospitals NHS Foundation Trust, Oxford OX3 9DU, UK; Mark.sullivan@ouh.nhs.uk (M.S.); Jacqueline.redgwell@ouh.nhs.uk (J.R.); 6 Radiology Department, Oxford University Hospitals NHS Foundation Trust, Oxford OX3 9DU, UK; ruth.macpherson@ouh.nhs.uk (R.M.); zoe.traill@ouh.nhs.uk (Z.T.); 7 Oxford Cancer and Haematology Centre, Oxford University Hospitals NHS Foundation Trust, Oxford OX3 9DU, UK; Mark.tuthill@ouh.nhs.uk; 8 Pathology Department, Milton Keynes University Hospital NHS Foundation Trust, Milton Keynes MK6 5DL, UK; Angus.Molyneux@mkuh.nhs.uk (A.M.); Elizabeth.Johnson@mkuh.nhs.uk (E.J.); Niveen.Abdullah@mkuh.nhs.uk (N.A.); 9 Department of Cellular Pathology, North Bristol NHS Trust, Bristol BS10 5NB, UK; Andrea.Taibi@nbt.nhs.uk; 10 Department of Cellular Pathology, Great Western Hospitals NHS Foundation Trust, Swindon SN3 6BB, UK; nikki.mercer1@nhs.net (N.M.); harry.haynes1@nhs.net (H.R.H.); 11 Institute of Biomedical Engineering, University of Oxford, Oxford OX3 7DQ, UK; Jens.rittscher@eng.ox.ac.uk; 12 Department of Computer Science, University of Oxford, Oxford OX1 2JD, UK; Jim.Davies@ouh.nhs.uk; 13 NIHR Oxford Biomedical Research Centre, Oxford University Hospitals NHS Foundation Trust, Oxford OX3 9DU, UK

**Keywords:** digital, pathology, testis, germ cell

## Abstract

Background: In this article we share our experience of creating a digital pathology (DP) supraregional germ cell tumour service, including full digitisation of the central laboratory. Methods: DP infrastructure (Philips) was deployed across our hospital network to allow full central digitisation with partial digitisation of two peripheral sites in the supraregional testis germ cell tumour network. We used a survey-based approach to capture the quantitative and qualitative experiences of the multidisciplinary teams involved. Results: The deployment enabled case sharing for the purposes of diagnostic reporting, second opinion, and supraregional review. DP was seen as a positive step forward for the departments involved, and for the wider germ cell tumour network, and was completed without significant issues. Whilst there were challenges, the transition to DP was regarded as worthwhile, and examples of benefits to patients are already recognised. Conclusion: Pathology networks, including highly specialised services, such as in this study, are ideally suited to be digitised. We highlight many of the benefits but also the challenges that must be overcome for such clinical transformation. Overall, from the survey, the change was seen as universally positive for our service and highlights the importance of engagement of the whole team to achieve success.

## 1. Introduction

### 1.1. Background

Digital pathology (DP), whereby whole slide images are created from glass slides (GS) to be viewed on computer workstations for purposes such as primary diagnosis, is now gaining significant traction in clinical practice, particularly in the United Kingdom (UK). Although a small number of laboratories have published their journey to large-scale or full digitization [1,2] and there are a few reports of the use of DP in multi-centre networks [3,4], there is surprisingly very little in the literature on the use of DP for highly specialist services requiring central review—despite the described clinical benefits of such a review [5] and the already common usage of DP for similar purposes in clinical trials for quality assurance [6].

### 1.2. Testicular Germ Cell Networks

Testicular germ cell tumour clinical services, including pathological diagnosis, are highly specialized and as such would seem to be an obvious specialty to benefit from DP and the subsequent ease of access to specialist review in-house and across a network. The UK National Institute for Health and Care Excellent (NICE) guidance “Improving Outcomes in Urological Cancer” recommended the establishment of supraregional specialized testicular cancer multidisciplinary teams (MDTs), serving a population base of 2–4 million and managing 50–100 new patients a year [7]. Patients with testicular cancer diagnosed by local urological multidisciplinary cancer teams should be referred to the specialist supra-network team and the diagnostic slides made available for review. Traditionally, glass slides have been sent from the local site to the central site via postal systems or hospital transportation systems. This manual process entails administrative time and effort to pack/unpack, book in and out of laboratories, and comes with an inherent risk of damage or loss of the slides. DP, having consistently shown benefits in efficiency and safety due to its virtual nature, has the potential to significantly add value to the working processes of these networks. Given this, as part of our transformation to a DP service in Oxford, we moved to a virtual or digital network for managing patients with testicular germ cell tumours in our region.

### 1.3. Aims

In this paper we describe and discuss our journey from June 2018 to March 2021, during which we achieved full digitization of our pathology laboratory, with an emphasis on how we transformed our existing supraregional network for testicular germ cell tumour reporting from analogue to digital. We outline the process, highlighting some of the challenges faced and using a survey-based approach to reflect on the user experience over this period. 

## 2. Materials and Methods

### 2.1. Aims

The aim of this paper is to describe and share our journey and experiences of transforming our traditional laboratory and network to a digital platform. We describe our experience of developing and validating this new way of working, and the importance of engagement of users in its success, the latter of which has been demonstrated through a series of surveys.

### 2.2. Pre-Deployment Assessment of Perceptions of Transition to and Utility of DP

Prior to the commencement of any DP deployment in the central site, we sought to assess the perception of pathologists and the wider multidisciplinary laboratory team who were part of a DP steering group, regarding the transition to DP in respect to their perceptions around potential benefits and challenges. This was part of the effort to engage the group to ensure that the process would be supported. An initial focus group explored the issues [8] and subsequently an online survey was circulated to the 13 members of the DP steering group using the SurveyMonkey survey tool (www.surveymonkey.co.uk; accessed on 13 October 2021). The survey was designed to assess both general considerations related to the transition to DP, and more focused questions addressing the predicted ‘benefits’ of adoption of DP.

### 2.3. Integration of DP Platform into Laboratories

The central laboratory in Oxford (Oxford University Hospitals NHS Foundation Trust; OUHFT) serves a large academic teaching hospital within which the consultant histopathologists practice as monospecialists or oligospecialists. The laboratory produces approximately 340,000 surgical histology and immunohistochemistry slides per year, together with 4100 extra-large slides, and handles around 40,000 referral slides.

The first Oxford scanner deployments were in the summer of 2018 with the installation of one Philips Ultrafast (UFS) scanner and one Philips Ultraversatile (UVS) scanner (Koninklijke Philips N.V., Amsterdam, Netherlands). The UFS has a 300-slide capacity and the UVS has a 60-slide capacity or can be loaded with 30 extra-large slides. A second UFS was deployed in summer 2019 and another in January 2020. In total, this was a 3 UFS, 1 UVS deployment which was anticipated to support the needs of the laboratory to 100% digitisation of surgical histology.

The program commenced with a pilot stage starting with testis/germ cell tumours, haematopathology, and medical renal biopsies; this was followed by remaining specialties coming online in a phased approach and accelerated by the coronavirus disease 2019 (COVID-19) pandemic. This staged approach has been previously described [9] and was taken to ensure that we first established the laboratory workflow and information technology (IT) adaptations necessary for DP locally and within the network, before rolling DP out further within the department. The specialties selected were referral heavy to develop the network and to demonstrate its utility. This process entailed the establishment of a barcoded workflow and the integration of our laboratory information system (LIS; Filemaker Pro) with the Philips Image Management System (IMS; Philips IntelliSite Pathology Solution 3.2, Image Management System 3.3 (L3); Koninklijke Philips N.V., Amsterdam, Netherlands). Post mortem and cytology slides were excluded from the full digital deployment at this stage, the latter in accordance with current UK Royal College of Pathologists (RCPath) guidance and recommendations [10].

**Figure 1:**
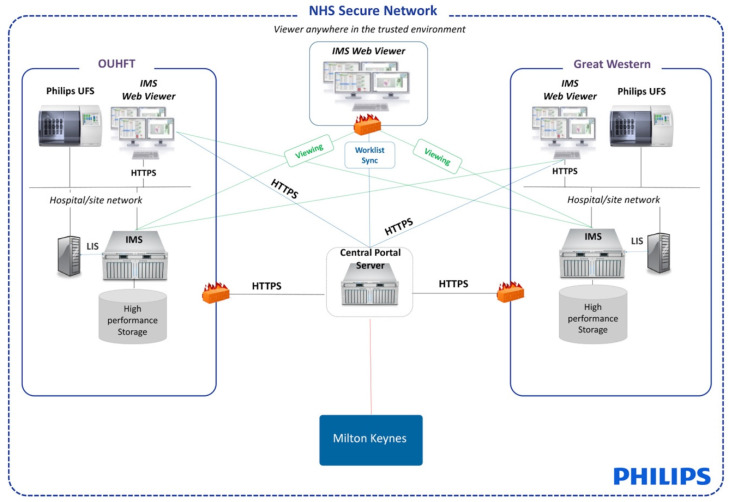
Architecture of solution design—Great Western Hospitals Foundation Trust (GWHFT) and Milton Keynes University Hospital Foundation Trust (MKUHFT) were bought online as part of the piloting of the supraregional network. MKUHFT has the same infrastructure as GWHFT, not shown here for simplicity. OUHFT is the central laboratory.

Scanners were deployed into the two other laboratories that form part of the existing Thames Valley Cancer supraregional germ cell tumour network (TVGCTN). A UFS was deployed into Great Western Hospitals National Health Service (NHS) Foundation Trust (GWHFT), Swindon and a UVS into Milton Keynes University Hospital NHS Foundation Trust (MKUHFT) in the summer of 2018. At GWHFT and MKUHFT, this enabled cases to be scanned for the supraregional germ cell MDT review in the local site and then digitally reviewed at the central site (Oxford). The solution design is shown in Figure 1. In addition, this enabled primary reporting of GWHFT testis cases at the central site to fill a staffing gap. Digital slide images were demonstrated at the germ cell MDT to the oncologists, radiologists, and other members of the MDT, enabling the clinicopathological correlation of cases. Access to the DP solution became particularly important during the COVID-19 pandemic, enabling pathologists to attend the meeting remotely and yet still show images of the cases to facilitate patient discussion.

### 2.4. Validation and DP Reporting of Cases

Three specialist germ cell tumour pathologists underwent the validation process for full DP reporting as described in the RCPath guidance [10]. This guidance outlines three steps—familiarisation with the DP platform, Stage 1 (retrospective set of cases covering the breadth of the pathologist’s practice), and Stage 2 (prospective digital case review with a glass check of relevant parameters). The case mix for validation will vary depending on the type of practice and cases encountered, e.g., specialist versus general practice. The RCPath guidance is not fully prescriptive, however, and the cases can therefore be selected by specialty teams; those we selected for testicular pathology for Stage 1 validation are shown in Table 1. As additional pathology specialities came ‘online’, consultants were given the opportunity to validate for digital reporting. Sign off was by the speciality lead and a pathologist familiar with DP if the speciality lead was not validated for digital reporting. We created DP mentors who were experienced digital users to guide other pathologists through the process. Pathologists were encouraged to share experiences of ‘potential pitfalls’ in digital diagnosis and other relevant learning during and after the validation Stages, with the experience of the pathologists taking part in the pilot of the project having additionally been captured in an online survey. Presentation of these experiences was included in several educational events, including a devoted study day for regional histopathology trainees. Importantly, a departmental DP Steering Group with a membership of pathologists and biomedical scientists was set up to ensure commitment to the delivery of the project and to monitor ongoing progress, addressing challenges and successes along the way.

Pathologists kept a record of all cases reported during the period of the study, including details such as primary reporting modality (digital/glass), confidence in diagnosis, number of slides in the case, date received/signed out, and any diagnostic discrepancies or challenges. Department-wide error logging was also key, particularly in the early stages, highlighting issues encountered by individual pathologists that formed trends that needed to be addressed, an example being the scanning of paler tissues such as adipose tissue.

### 2.5. Germ Cell Tumour Network—User Experience Surveys

Two separate online surveys were created on the SurveyMonkey platform, one for the multidisciplinary membership of the TVGCTN, and the other for the histopathologists, administrative team, and laboratory team, including those with a specific ‘DP champion’ role, with direct involvement in the digitisation of testis and germ cell tumour cases in OUHFT, GWHFT, and MKUHFT.

#### 2.5.1. Survey 1: The Impact of Access to DP in the Setting of the TVGCTN, on the Laboratory Staff, Administrative Staff, and Histopathologists within OUHFT, MKUHFT, and GWHFT

The primary outcome of this survey was to assess the opinions of the pathologists, and the impact of our transition to, DP in the setting of the TVGCTN. Specifically, we aimed to assess the challenges to the implementation and roll-out of DP across the network, as well as the perceived benefits and any potential wider benefits that may be anticipated going forward. Analysis of the survey results was intended to highlight areas that could potentially be improved upon during the planned wider transition to DP across the network, and to ensure ongoing engagement and support of those involved in the process.

The survey was divided into a set of seven general questions applicable to all respondent groups, and then three sets of questions specific to those working in the laboratory team, the administrative team, and the pathologists. The respondents were asked to limit their answers to the specific questions relevant to their role. The general questions included those around perceived benefits of DP including those specific to the TVGCTN and the patients being managed within this network. There was opportunity for free text answers, and respondents were asked for (anonymous) examples where applicable.

#### 2.5.2. Survey 2: For the Members of the TVGCT MDT

The primary outcome of this survey was intended to assess opinion amongst the multidisciplinary membership (non-pathologist) members of the supraregional MDT network regarding the introduction of DP, to identify any perceived challenges, and specifically to explore potential wider benefits of DP in this setting beyond that of the diagnostic reporting of cases. All members of the TVGCNT MDT were invited to respond to the survey. The survey was composed of 12 questions, with opportunity for free text responses. The questions explored current awareness of the availability of DP, of its impact, and of potential wider benefits to the MDT meetings, to patients, and in terms of education.

## 3. Results

### 3.1. Pre-Deployment Assessment of Perceptions of Transition to and Utility of DP

Engagement of the pathologists and the wider team within the department at OUHFT was an important aspect of the journey to DP. The focus group at the outset provided an insight into the general perceptions of the group [8]. There were nine respondents to the subsequent online survey of the pilot group (seven histopathologists), which revealed the aspirations of the group in respect to the promise of DP to improve aspects of patient safety, diagnostic workflow, workforce planning, and service quality, very much in line with those reported by others [11]. The survey highlighted concerns around ease of request for a second opinion for cases and the potential impact of this on workload, and also raised concerns over the clarification of governance around patient data, and issues related to data storage capacity. These issues were included in steering group discussions over the subsequent months. The steering group for the central site was superseded by a network wide user group with the governance aspects subsequently becoming part of the standing agenda for the departmental governance group meetings.

We also considered the perceptions of those training in histopathology of the utility of DP and of the transition process, particularly given the variability of access to DP within the training centres in the region, given that general utility of DP is currently limited to OUHFT [12]. It was evident from this work that trainee pathologists’ needs were an important consideration during the rollout, particularly from the training and validation aspect, and the potential need for them to continue reporting on GS as well as DP.

### 3.2. Integration of DP Platform into Laboratories

The central laboratory successfully achieved full digitisation (scanning 100% of surgical histology workload) in summer 2020. Four scanners were sufficient to handle the slide volume, but there was little spare capacity. We thus concluded that to provide this additional buffer we would purchase a further scanner. Other issues encountered included occasional failure of synch between the IMS and the LIS and extra-large (XL) slide labels not being readily viewable in the IMS. The latter was recently addressed with a software update and underscores the importance of IMS/LIS integration in this setting to ensure that all slides are available for viewing. It took longer than anticipated to establish links between the IMSs of each site, which was a reflection of the operational, organisational, and governance complexities across a three-site network rather than technical issues per se. Once on-site servers were full at the central site, a cloud archive was established (Microsoft Azure). Scanning at MKUHFT had to temporarily cease when their on-site server storage became full and further funding to expand it as part of the upscaling AI Centres funding program was awaited.

Slide recall from the archive per year for Oxford was evaluated to model the predicted costs of image recall from the cloud store; this was 2249 slides recalled per year for diagnostic purposes and 9168 for research purposes (including for clinical trials). It is too early for us to formally evaluate the impact of a cloud-based digital archive on previous case retrieval, although subjectively this system is simpler and more time efficient than glass slide recall. As per the RCPath and Institute of Biomedical Science guidance, both the GS and digital images are retained for two accreditation inspection cycles (around 8 years) [13], a situation which is unlikely to change and which is an important consideration for laboratories planning the transition to DP.

### 3.3. The Impact of COVID-19

Part way through our deployment in March 2020, WHO declared COVID-19 to be a global pandemic. Our platform provided a useful solution to some staffing challenges related to self-isolation and shielding and additional guidance based on a risk-mitigated approach to use of the platform was created for the department. This was based on the principles outlined in the RCPath emergency guidance for remote DP working [14]. The COVID-19 pandemic accelerated the uptake of DP in our department [15], with pathologists widely utilising the platform in a variety of ways falling short of full digital diagnosis, even if not fully validated (using a risk-mitigation approach), and with greater numbers opting to commence the validation process sooner than they had anticipated. COVID-19 also resulted in a shift to MDT meetings being conducted virtually with Microsoft Teams, something which was utilised by pathologists who were also then able to prepare for MDTs digitally and live screen share digital slides with other MDT meeting attendees.

In addition to an impact on pathologist uptake of DP, there was a direct impact on the process of the roll-out of DP within the department as the wider utility of the system highlighted deficiencies in the workflow. Many of these aspects were being addressed, but the process was accelerated to facilitate remote working; for example, the scanning of referral letters and paper request forms bearing the clinical details for a case was implemented to ensure these were accessible alongside the digital slides on the IMS for remote review by pathologists working from home. Similarly, the workflow to ensure that macroscopic images of a case were available on the IMS was finalised. Routine scanning of all referral cases was recognised as a need but entailed additional considerations around slide labelling and administrative workflow adaptations, but again this aspect of the workflow was brought forward and addressed, and we saw routine scanning of all referral cases at the end of summer 2020.

The COVID-19 pandemic does however, mean that general assumptions about improved workflow efficiencies due to full digitisation cannot be formally assessed for this study as there are too many variables/confounding factors, including increased remote working. However, the general impression is that workflow improvements have been seen, especially access to second opinion/double reporting [16].

### 3.4. Validation and DP Reporting of Cases

Whilst the department followed the RCPath guidance [4] in respect to validation for DP reporting, this was adapted slightly to ensure that we were able to fulfil the recommendations of our local governance group. An electronic record by way of a log was created for the validation process, within which a pathologist was able to review the digital cases selected for the Stage 1 validation and document their interpretation of the case and the confidence on DP assessment vs. glass. There was a separate electronic log for Stage 2 of the process. When a pathologist was confident that they had completed the validation, it was possible to create a download of these records for review for the ‘sign-off’ process.

During Stage 2, discrepancies were considered to be potentially clinically important where no glass check would have been performed. In relation to testis cases across the three pathologists, there was only one example of this which was related to a subtle pagetoid spread of the tumour within the rete not being seen on digital but noticed on glass. This was categorised as a minor error in a supplementary parameter (RCPath error classification) [17]; pagetoid spread has no clinical implications and is a non-core data item in the RCPath dataset for the reporting of testicular neoplasms. One comment common to the three pathologists was related to seminoma vs. solid pattern embryonal carcinoma being more difficult on digital. Other areas of challenge identified were variable between the pathologist; for example, Pathologist 1 described feeling that architectural features such as hilar soft tissue invasion were more easily assessed on digital due to the low power overview of slides that is possible with digital, and that they had a high confidence for lymphovascular invasion (LVI) assessment on digital, which was different to the experience of Pathologist 2, who felt that staging (e.g., hilar soft tissue invasion, LVI) was more challenging initially, though they became more confident with this. Pathologist 1 noted screening background tissue for germ cell neoplasia in situ (GCNIS) or small areas of seminoma was more difficult initially, but they became more confident as time went on, while Pathologist 2 felt confident with background changes. Pathologists were asked to specify their preferred reporting mode (see Table 2) and there were inter-personal differences in confidence and preference.

As an example, Pathologist 1, the supraregional lead for the service, reported 92 digital germ cell tumour or testis cases in the period of October 2019 to March 2021 (27 while in Stage 2, the remainder were fully digitally reported), including benign and malignant entities. These included 38 GWHFT cases primary reported, 20 cases arising in the central site, and 34 cases as second opinion/MDT review. Forty-eight of these cases had been scanned in GWHFT or MKUHFT. There was a full range of benign and malignant entities, including germ cell tumours, sex cord stromal tumours, metastatic germ cell tumour biopsies, and mediastinal germ cell tumours. A small number of cases during this same period could not be reported digitally by Pathologist 1 due to various issues: in two cases slides had not been scanned before being sent to the central site and in one case this was because the server at one of the local sites was full, six had technical issues (e.g., slides not being viewable on the portal of another site), and four cases could not be digitised due to a scanner being down and a lack of capacity. Pathologists 2 and 3 reported an additional 31 cases digitally during the same period, the smaller case numbers than Pathologist 1 reflecting pro-rata case distribution and periods of leave, making a total of 123 germ cell tumour/testis cases reported digitally in a 17-month period (which is a typical number expected for this service in that timeframe).

### 3.5. Germ Cell Tumour Network—User Experience Surveys

#### Survey 1: The Impact of Access to DP in the Setting of the TVGCTN, on the Laboratory staff, Administrative Staff, and Histopathologists within OUHFT, MKUHFT, and GWHFT

There were nine respondents to the survey sent out to the departments, representing the views of respondents across a range of roles: two biomedical scientists (BMSs), three administrative/secretarial staff, one laboratory manager, one DP champion (DP champions were pathologists or BMSs depending on the site), and three histopathologists. All general questions were answered in full, except for one which received 8/9 responses. All respondents were aware of the introduction of DP within the GCT network, and all regarded this as a positive step. All respondents agreed that access to DP offered the potential to reduce administrative burden, reduce potential delay in case transit into OUHFT, and improve the speed of access to the cases for specialist review, with improvements in the ease of access to previous histology. Free text comments regarding direct patient benefit included a comment that DP had already facilitated urgent specialist review of cases for two patients that had directly impacted on patient management, as the more rapid diagnosis enabled an early decision to treat with chemotherapy. There was a separate comment as to the benefit to pathologists working in separate centres through the ability to share cases through DP. In terms of disadvantages, two commented on cost (but without specific detail) and one that DP was an additional step in the workflow process.

### 3.6. Impact of the Introduction of DP on the Department

**Figure 2:**
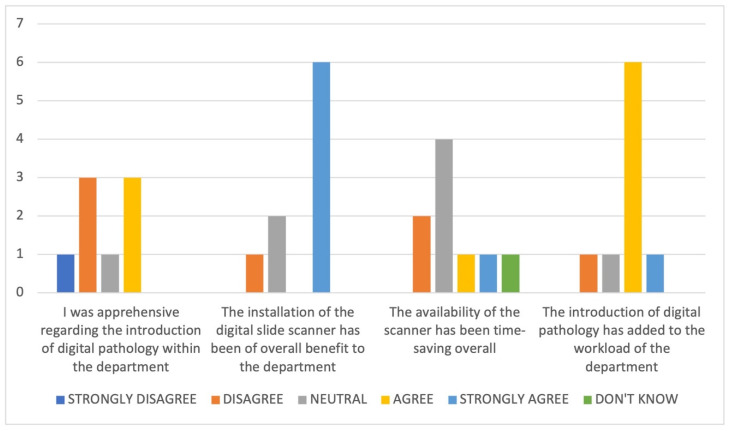
Collated responses in relation to general considerations around the availability of DP within the departments in the TVGCTN. Questions in relation to DP relevant to each of three specific roles within the department were asked to explore issues including the perception of impact on workload. These are presented according to role in Table 3.

The survey included general questions to explore perceptions related to the introduction of DP within the department, and particularly around department workload (see Figure 2 and Table 3). From the responses, it is apparent that whilst the majority (across roles) perceive DP to be of overall benefit to the department (6/9), there is a perception that having access to DP has added to the workload of the department (7/9).

These are examples of some of the challenges raised in the free text comments (not given verbatim):

- The new process has taken time to adjust to, with things taking much longer;
- Time is needed to clean and prepare slides for scanning;
- There is no real advantage whilst the GS are still being posted;
- An issue with the link between one of the hubs’ peripheral sites and the central site at OUHFT created problems with DP case review;
- The workflow and expansion of the service is a work in progress but will be of benefit in the long term;
- Workflow issues such as out of focus images and missing slides and issues such as storage, training, integration, and cost need to be resolved.

Three pathologists responded to the survey; however, all three were based within the central site at OUHFT and the results therefore did not incorporate the opinions of those based within the centres referring into the TVGCTN. All three pathologists were reporting GCT cases on DP and felt that this was a positive experience. Two (of three) felt that overall, the turnaround time for cases being received for review for the TVGCTN MDT was reduced through the availability of DP, and all three felt more comfortable in the knowledge that the slides for these cases had been digitised as well as being posted. All three pathologists utilised DP for second opinion (giving and requesting) from colleagues within the department and felt that this was easier than on GS, but for one (of three) this did not mean that they were more likely to request a second opinion. The three pathologists all felt that the demonstration of histology images for the purpose of the MDT was easier digitally, and for the two (of three) who had utilised DP for this purpose, they agreed that it had benefitted MDT discussion.

#### Survey 2: The members of the TVGCT MDT

**Figure 3:**
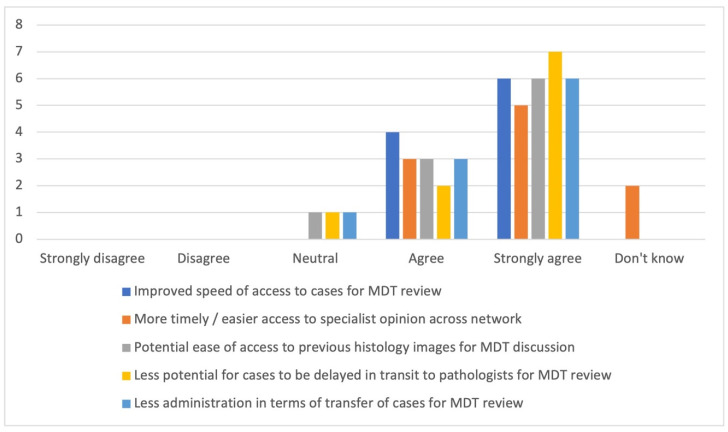
Collated responses in relation to general considerations around the availability of digital pathology within the Thames Valley Germ Cell Tumour Network.

There were 10 respondents to the survey: three histopathologists, three oncologists, two MDT co-ordinators, one radiologist, and one specialist nurse. All but one (of 10) were aware of the introduction of DP at OUHFT, and all regarded this as a positive step. The overall opinion was entirely positive in regard to the potential benefit from DP in relation to the ease of access to specialist opinion and for the review of cases, and to the speed of access and the potential for a reduction in administrative burden (see Figure 3).

In general, the histopathologists were able to cite more examples of potential advantages and challenges with DP. In terms of general advantages, they cited examples of expedited expert opinion facilitating more timely access to treatment, improvements in turnaround time during enforced (COVID-related) remote working, ease of review of cases for MDTs, and ease of access to opinion between pathologists in the same centre and potentially in other centres. The latter aspect was, however, also noted to be a potential challenge in relation to the impact on the workload of the specialist pathologists, and the other main challenges identified included the technical aspects of DP set-up and the potential for DP reporting to be slower.

**Figure 4:**
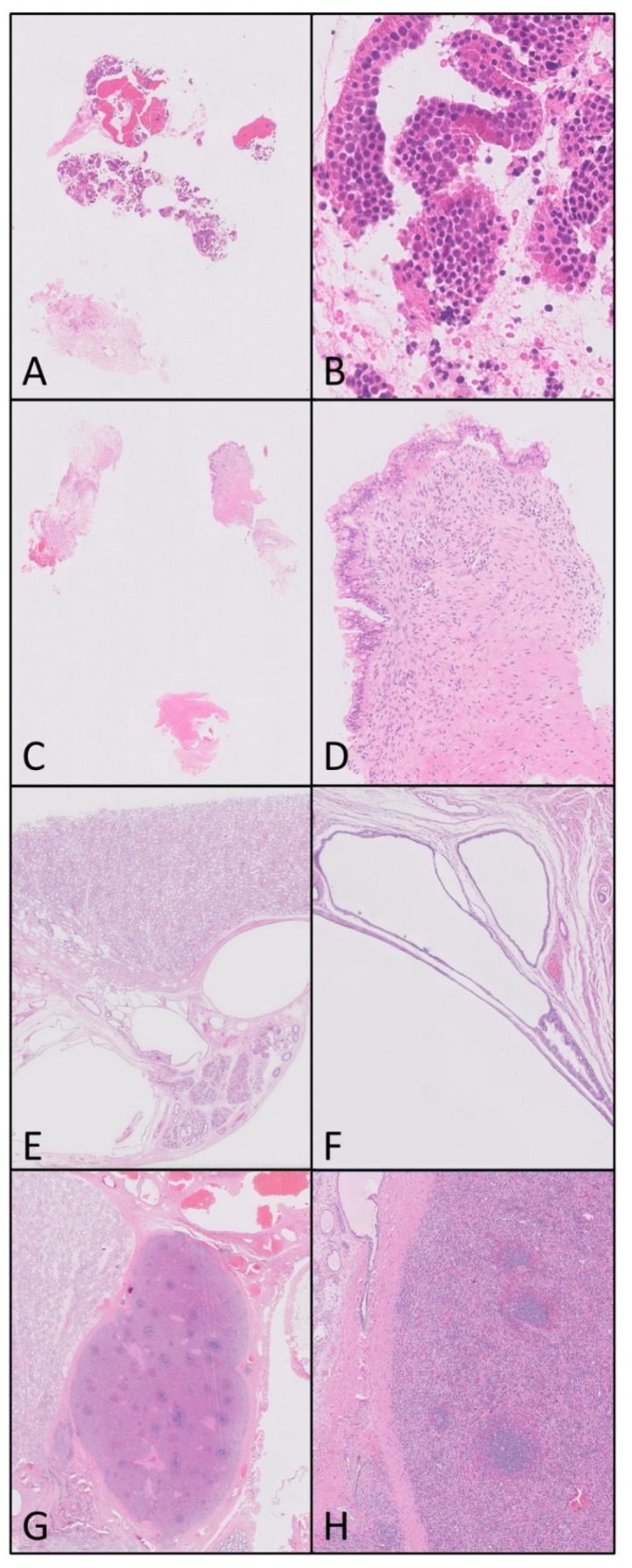
Examples of cases where displaying DP images aided or facilitated the discussion at the multidisciplinary team meeting (MDT). (**A**) A testicular mass biopsy that was shown at the MDT to demonstrate the limited nature of the material present and explain why reaching a definitive diagnosis was difficult. (**B**) A higher-power view of this same tumour which was thought to most likely be in keeping with a carcinoid tumour after extensive additional investigations. Here the problem included the difficulty in excluding or confirming a background teratoma or other germ cell components. (**C**) A biopsy from a 9 cm retroperitoneal tumour; although it shows features of a teratoma (thought to have a primary testicular clinically), the (**D**) higher-power view again demonstrated at the MDT the uncertainty of how representative this small amount of tissue is of the main lesion. (**E**,**F**) A benign cystically dilated rete testis which was shown at the MDT to correlate directly with imaging and to confirm the diagnosis (note that bottom left area of missing tissue in (**E**) is a microtomy artefact). (**G**,**H**) A rare diagnosis of splenogonadal fusion which was shown at MDT (higher-power) for the educational interest of the team members.

Asked about the availability of DP for the visualisation of pathology during an MDT meeting, the responses were overall positive, although mixed. Seven (of 10) felt that DP images were easier to appreciate than projected GS images (which could not have been achieved remotely), six (of 10) regarded the demonstration of histopathology during the MDT as helpful in understanding the pathology report, and eight (of 10) felt it was beneficial when reviewed in conjunction with the radiology. Figure 4 illustrates a number of examples of germ cell tumour and testis cases where digital images were shown at the MDT. However only three (of 10) felt that they wanted to see relevant pathology demonstrated at the MDT, with six (of 10) remaining neutral on this (including one histopathologist).

One clinical member of the team commented:

*‘… I still feel it is important to be able to review images at the MDT and particularly useful for me as a clinician to have interaction with pathologists and understand the pathology. Impressive to see the digital images.’*

The response to the potential for educational resources utilising DP was also positive, with all of the clinical (non-histopathology) members of the team suggesting that this would be of interest.

Regarding potential patient benefit from the availability of digital images during a consultation to demonstrate to patients, none of the oncologists felt it would generally be of benefit, even if the image was annotated in advance, and one comment suggested that this may in fact potentially complicate discussions, especially if the patient asks a pathology-related question.

## 4. Discussion

### 4.1. The Move to DP

The move to DP is now gaining pace in the UK, and this has been recognised at a national health level. DP was highlighted in the UK Government’s Industrial Life Sciences Strategy for its potential to create substantial efficiencies by an increasingly virtual service and facilitating network working [18]. The potential quality and efficiency benefits of DP have been previously described [11,19], and it is beyond the scope of this paper to cover them here again in detail. For patients, the benefits may include the potential to access expert review in a timelier fashion. Diagnostic concordance between DP and glass has been shown to be 98.3% on systematic review and meta-analysis [20]. The COVID-19 pandemic has highlighted the potential for DP to help maintain pathology services in times of crises, for example by enabling remote working [16,21], and has served as a catalyst to DP uptake [15,22].

Our transformation to DP in Oxford has been driven by national funding. In 2018 the UK Government created 5 Artificial Intelligence (AI) centres of excellence and one of those centres, PathLAKE, includes our centre (OUHFT), which now has a fully digital histopathology laboratory, one of the first few in the UK to reach the milestone of scanning 100% of its surgical histology slides and referrals, and achieving the International Organisation for Standardization (ISO) 15189 accreditation (Medical Laboratory Accreditation) with the UK Accreditation Service (UKAS) for DP. In our experience, one of the earliest successes of DP was using the platform for the primary diagnostic reporting of testicular specimens and deploying scanners in two partner Trusts (hospital organisations), providing digital infrastructure for the TVGCTN.

### 4.2. The Benefits of Supraregional Working in Testicular Germ Cell Tumour Management

The benefits of supraregional (central) review of testicular germ cell tumour pathology have been previously described with emphasis on the importance of the concentration of pathologist experience in assessing these relatively rare tumours [23]. Although there is a much better awareness of diagnostic pitfalls than at the inception of these networks, contemporary review still demonstrates the value of supraregional review.

### 4.3. Digitisation of Oxford and the Supraregional Network

Although, as we have illustrated, DP has been successfully achieved, both for primary diagnostic reporting and to facilitate MDT networking and specialist review, challenges have been encountered along the way. We hope that sharing our experiences will be helpful for others aiming to create a DP specialist network or aiming for full digitisation for diagnostic pathology purposes. We describe here the full digitisation of a central laboratory and the deployment of slide scanners to two further centres in the Thames Valley supraregional germ cell tumour network and demonstrate the feasibility of reporting these cases over a digital platform. Digitisation of the TVGCTN was anticipated to offer advantages to our network which we feel have been achieved, and the feedback from both the laboratories involved in the network and the MDT team has been very positive.

One aspect of network access to DP that was anticipated to be a potential challenge from the pre-digital survey and the focus group was the perception that there could be an increase in requests for second opinions, both within the local centre at OUH and between centres (unpublished data). However, anecdotally this does not appear to have been the case. The pathologists reporting testis cases at OUH suggested that they would not be more likely to seek a second opinion just because DP made it easier to do so but have commented consistently that access to DP does make second opinions/double reporting easier and timelier, which has a positive impact on patient care.

Whilst it has been possible to successfully establish DP connectivity across centres within the setting of a supraregional MDT network on which all sites are using the same hardware and software, there is not currently a general solution for referral practice between centres across NHS networks [24]. In a centre with 22,000 external referral slides per annum, of which only some will be related to a networked MDT, we would like to see the development of a facility to share digital slides from other centres, both in terms of ease of offering a second opinion, and for seeking further opinion, particularly in super-specialised areas of pathology. There are, however, important additional considerations for the set-up of such a system, which include information governance and data sharing/security issues. A Dutch group has recently published an overview of a vendor-independent platform developed for use in the Netherlands for connectivity between centres [25]; however, at the present time such a system is not widely available in the UK. The potential benefits of the availability of such a platform are vast, including education and research applications, and advances in the development of such a system would be welcomed.

It is recognised that the introduction of technology into healthcare is disruptive, and attention to the factors that impact the success of any such implementation is key [26], as is engagement with all departmental staff involved. There are other parties who also need to be engaged to facilitate the process and its long-term success, such as internal governance bodies, IT support teams, and information governance teams.

Whilst the laboratories within the network uniformly supported the transition to DP, there was a recognition within the feedback we received that the process was an additional demand on time, and that it required attention to workflow changes. Whilst there was also a perceived awareness that there was a learning curve necessary for the implementation of DP, it was also felt that the end reward was worthwhile, both for patients and for the multidisciplinary laboratory team involved. However, an appreciation that this transitional process entails additional time and effort is important; commitment from the whole team is necessary for success. Some of the additional effort currently is attributed to the scanning of referral cases for the supraregional service from centres other than MKUH and GWHFT which do not currently have scanners, and thus slides are scanned on receipt at OUHFT.

Specifically in relation to the DP transition for GCT, whilst all three pathologists were positive about the step and were supportive of the process, we identified differences in the areas they found to be more challenging and in their initial confidence. This was in part related to the number of cases reported; GCT are not a large proportion of the workload, and as such exposure to these cases even in a specialist centre where we would expect to see 10 cases per month (split between three pathologists), is not huge in comparison to other subspecialties. Varying levels of confidence with DP are to be expected and will depend on various factors such as personal factors, pathology experience, and prior experience with digital.

To date few of the discrepancy studies evaluating the diagnostic concordance between glass and digital include any testicular cases, and they typically focus on the primary diagnosis of higher volume specialties such as skin and gastrointestinal (GI) pathology. Given that much of testicular pathology practice is related to the central review of referrals rather than primary diagnosis and that these cases are concentrated in certain centres, it is likely that this practice was therefore not reflected or was under-represented in these centres undertaking validation studies. One DP study at a large academic centre looked at a typical day’s workload and did include four testicular cases; one of these accounted for a diagnostic discrepancy whereby a yolk sac tumour component had been seen on glass but not on digital in a mixed germ cell tumour with a majority of seminoma [27]. Therefore, whilst there is currently very little literature on potential diagnostic discrepancies between DP and glass in testicular pathology, from the existing literature it appears that on the whole the pitfalls that seem to affect other specialties such as difficulty in assessing the grade of dysplasia or identification of mitoses or micro-organisms would not be particularly relevant. However, documentation of any potential features causing discrepancy is essential during the transition to DP and sharing of this experience is important amongst pathologists.

We found that pathologist review of relevant parameters in Stage 2 on glass for testicular germ cell tumours can vary widely depending on what the question is. If you are looking to exclude LVI in a germ cell tumour, then you may need to review all the tumour containing slides; however, if you find LVI on the first slide, then further review for this parameter can be more ‘light touch’. Similarly, reviewing a seminoma with a large number of slides to exclude a small element of non-seminoma again warrants a more detailed approach. Although some areas were initially found to be more challenging on digital at first by some pathologists, pathologists found different aspects to be more difficult; for example, one pathologist found screening background testis tissue to be more difficult, while another found it easier. Importantly, following the validation process, no consistent diagnostic difficulties have been identified, although the purpose of this study was not a formal one of diagnostic concordance. Our department has a requirement for an ongoing audit of cases reported digitally, and we maintain a regular dialogue on DP amongst our specialty team to ensure we continue to share experience.

### 4.4. COVID-19

The COVID-19 pandemic accelerated the uptake of DP in our laboratory, and digitally enabled care should now be seen as a core component of service planning [16]. The changing clinical landscape and working patterns mean that capturing information formally on efficiency savings that have been reported in the literature in some studies, but not by others [4,27,28] due to DP during this period, which is already complex, was impossible during this phase. Quality benefits are easier to describe, and have been highlighted here, but may be qualitative. The COVID-19 pandemic also highlighted the possibility of using DP to remotely work for some of the time, and we have previously reported the positive impact that this had on maintaining diagnostic services during this (ongoing) period of crisis [18]. Traditionally, pathology has been an on-site activity with occasional remote working. DP has a potentially significant role to play in climate change and reducing the movement of people and slides around the country and reducing the inequity of access to services.

### 4.5. Feedback on the DP Experience of the Wider TVGCT Network

From the surveys conducted amongst the multidisciplinary membership of the TVGCT MDT, and the departments within the three centres networked with DP, it is clear that the introduction of DP into the network is seen as a positive step. There were already direct patient benefits from DP highlighted, with examples of patient care being positively impacted by more timely diagnosis through the availability of expert opinion more quickly than if GS were moved around. DP was also recognised by the MDT team to be beneficial to MDT discussion, with 6/10 finding the demonstration of images useful, and 7/10 agreeing that DP was a better format for the demonstration of images (see Figure 4). It was particularly useful to show images in cases which were diagnostically challenging or required correlation with the imaging findings to aid understanding. DP was also felt to be appealing for educational purposes, and in direct response to the survey feedback we plan to develop a DP facilitated educational session for non-histopathologists.

### 4.6. Future Considerations

Whilst our laboratory successfully underwent a UKAS extension to scope inspection for DP, the other sites in our network do not have the specific accreditation extension for DP and thus our default position is currently to review the relevant parameters of GS of cases scanned at the other sites. Once these sites have UKAS extension to scope, for which we can cascade our experiences to expedite this process, we would only need review the digital images of the case for diagnosis, unless there was a specific reason to request the GS, at which point we will fully realise the benefits of DP in this context.

We acknowledge that during the term of this study, while we did digitise the central site, we did not digitise all the sites in the TVGCTN or create fully digital sites at MK or GWH, but the pilot deployment at these two other sites demonstrates the potential for the whole network. Funding has currently been successfully sought to fully digitise these two sites and one other, which represents our local pathology network. Certainly, our learning will facilitate a smoother process as we work to expand DP access now at a larger scale more widely in the region, particularly in the realm of governance and infrastructural issues.

The deployment of DP provides the infrastructure on which to unlock future benefits that may be afforded by the potential to allow standardised reporting and AI algorithms, which can either support pathologist reporting or provide novel insights into disease biology. While algorithms with European Conformity in vitro device (CE IVD) approval enabling diagnostic use exist in other tumour types, such as prostate biopsy reporting [29,30], very little has been published on the potential use of AI in testicular germ cell tumours. One report focused on the automated detection of LVI by AI [31] and another on the prognostic impact of tumour infiltrating lymphocytes [32]. In general terms, these tumours, being relatively rare, have limited datasets for algorithm building and marked heterogeneity due to mixed tumour typing makes the training and validation of AI more challenging; thus, such tools remain at relatively early stages in the roadmap to clinical deployment [33]. The centralisation of digital images of cases across a specialist network has the potential to accelerate potential development in the understanding of the biology of these tumours and the potential then for the future use of AI.

## 5. Conclusions

In summary, DP can be utilised across supraregional and specialist services to create highly effective systems with the potential for improved efficiency, virtual but closer working with regional colleagues, and ultimately a better system for patients. Importantly, the infrastructure for deployment of AI, where further benefits are likely to be seen, is in place.

## Figures and Tables

**Table 1:** Cases in Stage 1 of digital pathology (DP) validation.

Details (Tissue Type/Specimen Type/Preparation/Stain).
Mixed germ cell tumour (embryonal carcinoma, yolk sac tumour, teratoma) Rete testis stroma invasion Hilar soft tissue invasion LVI
Leiomyosarcoma
Spermatocytic tumour Referral
Leydig Cell Tumour Various IHC (calretinin, CD99, inhibin, MelanA, panCK, OCT3/4, PLAP, CD30, AFP, Ki-67)
Seminoma Rete testis stroma invasion Hilar soft tissue invasion Spermatic cord invasion IHC (OCT3/4, C-Kit, D2-40, LCA, CD30, panCK)
Seminoma Tumour smearing artefact
Regressed germ cell tumour OCT3/4 IHC
Mixed germ cell tumour (seminoma, embryonal carcinoma) GCNIS
Mixed germ cell tumour (seminoma, embryonal carcinoma) LVI Rete testis stroma invasion GCNIS Various IHC Comment: Challenging case was originally said to have hilar soft tissue invasion but was later revised to LVI in hilar soft tissue only.
Benign: Atrophy
Mixed germ cell tumour (teratoma, embryonal carcinoma, yolk sac tumour) LVI
Seminoma GCNIS Separate soft tissue deposit in the cord (M1) Comment: Described as no unequivocal LVI, therefore challenging.
Diffuse large B-cell lymphoma
Seminoma LVI in cord Comment: Difficult case—differential diagnosis of LVI in cord versus a soft tissue deposit (M1).
Seminoma Partial orchidectomy
Mixed germ cell tumour (embryonal carcinoma, yolk sac tumour, teratoma) LVI
Seminoma Hilar soft tissue invasion Comment: LVI assessment very difficult, needed IHC (D2-40).
Mixed germ cell tumour (yolk sac tumour, teratoma, seminoma) Comment: Various IHC needed to clarify tumour components.
Seminoma Comment: Challenging case. Focal rete testis stroma invasion added after MDT review.
Benign: Abscess
RPLND: Viable yolk sac tumour and post chemotherapy teratoma
Benign: Infarct/torsion Referral
Partial orchidectomy Choriocarcinoma Embryonal carcinoma Teratoma Seminoma Yolk sac tumour Referral Mega slides
Metastasis Biopsy Seminoma Various IHC Referral
Regressed germ cell tumour—scar Referral Challenging case Very focal GCNIS
Mediastinal biopsy Seminoma IHC

**Table 2:** Results of Stage 2 pathologist views. **A**—Pathologists’ preferred method of reporting; digital, glass, or no preference (either). **B**—Pathologist confidence scores on digital reporting; scoring system ranged from 1 to 7, with 1 being the least confident and 7 being the most confident. **C**—Pathologists’ confidence scores on glass reporting.

**A**				
Pathologist	Preferred Method of Reporting—Digital (%)	Preferred Method of Reporting—Glass (%)	Preferred Method of Reporting—Either	Not Recorded (%)
1	67	12	19	2
2	0	24	76	0
3	100	0	0	0
**B**				
Pathologist	Confidence Score 4 (%)	Confidence Score 5 (%)	Confidence Score 6 (%)	Confidence Score 7 (%)
1	1	1	8	88
2	0	35	53	12
3	0	0	18	82
**C**				
Pathologist	Confidence Score 4 (%)	Confidence Score 5 (%)	Confidence Score 6 (%)	Confidence Score 7 (%)
1	0	1	10	87
2	0	0	35	65
3	0	0	9	91

**Table 3:** Perception of the impact of DP—opinions of those involved in slide scanning and administration.

**Questions to Those Involved in Slide Scanning**						
	**Strongly Disagree**	**Disagree**	**Neutral**	**Agree**	**Strongly Agree**	**Don’t Know**
Scanning of GS has been easy to introduce within the department	0/5	1/5	1/5	3/5	0/5	0/5
Scanning of GS has not impacted significantly on my workload	0/5	2/5	2/5	1/5	0/5	0/5
Scanning of slides is efficient	0/5	1/5	1/5	2/5	1/5	0/5
Scanning of slides fits into a lean workflow	0/5	1/5	3/5	0/5	1/5	0/5
I understand how my role to enable scanning of these cases fits into the overall strategy for digitising this TVGCTN	0/5	0/5	1/5	3/5	1/5	0/5
The training I have received has been timely and sufficient to give me confidence in this role	0/5	1/5	1/5	1/5	1/5	1/5
I would like additional training for this role	1/5	0/5	1/5	1/5	0/5	0/5
**Questions to Those Involved in the Administration of Slides for MDT Referral**						
Scanning of GS has not impacted significantly on my workload	0/4	1/4	2/4	1/4	0/4	0/4
Now that slides are scanned, packing and sending cases in the workflow is less pressurised as pathologists can very quickly access the slides digitally	0/4	2/4	1/4	0/4	1/4	0/4
I understand how my role to enable scanning of these cases fits into the overall strategy for digitising this TVGCTN	0/4	0/4	1/4	2/4	1/4	0/4
I feel more comfortable in the knowledge that cases are digitally scanned as well as physically posted to another department	0/4	0/4	1/4	2/4	1/4	0/4
The availability of DP has made my role (in the TVGCTN setting) easier	1/3	0/3	2/3	0/3	0/3	3
